# Performance of molybdenum vanadate loaded on bentonite for retention of cesium-134 from aqueous solutions

**DOI:** 10.1007/s11356-023-26607-z

**Published:** 2023-04-06

**Authors:** Mariam Ghaly, Mohamed Ragab Abass, Zakaria Ali Mekawy

**Affiliations:** Hot Laboratories, and Waste Management Centre, Egyptian Atomic Energy Authority, Cairo, 13759 Egypt

**Keywords:** Cs(I), Bentonite, Kinetic, Isotherms, Thermodynamic, Recycling

## Abstract

This article studied the sorption behavior of Cs(I) ions from aqueous solutions onto molybdenum vanadate@bentonite (MoV@bentonite) composite. MoV@bentonite has been fabricated using the precipitation method and was characterized by different analytical tools including, FT-IR, XRD, and SEM attached with an EDX unit. The sorption studies applied on Cs(I) ions include the effect of contact time, pH, initial metal concentrations, ionic strength, desorption, and recycling. The experimental results revealed that in the adsorption process carried out after equilibrium time (300 min), saturation capacity has a value of 26.72 mg·g^−1^ and the sorption of Cs(I) ions is dependent on pH values and ionic strength. Sorption kinetic better fit with the pseudo-second-order model; sorption isotherms apply to Langmuir, Freundlich, and Dubinin-Radushkevich (D-R) isotherm models. Data of thermodynamic parameters indicate that sorption is spontaneous and endothermic. Recycling experiments show that MoV@bentonite could be used for 7 cycles and the best eluant for the recovery of Cs(I) ions is 0.1 M HCl (76.9%). All the obtained data clarify that MoV@bentonite is considered a promising sorbent for the sorption of Cs(I) ions from aqueous solutions.

## Introduction

During the fission reactions, several corrosive effluents containing radionuclides either short or long-life elements are produced. Removal of these radionuclides from radioactive waste has recently gained great attention as they became more profound with increasing radiation activities. Cesium is one of these pollutants, and it is found in radioactive waste from industry and medicine, as well as research and nuclear facilities, necessitating its separation from aqueous solution. In addition to some toxic organic compounds that are suspected to be carcinogens or allergens, these compounds can cause fatal damage (Abdel Rahman et al. 2019; Lin et al. 2020). Cesium is usually present in water in the cationic form with high mobility and solubility rates; thus, it can be mobile and bioaccumulate in animals and plants which affects the food chain (Noli et al. 2021). Although human health is hardly affected by stable cesium isotopes, the ingestion of Cs-134 leads to a serious disorder of human health and even death; the main reason is due to its chemical similarity to sodium (Na) which leads to its precipitation in the soft tissues in each part of the body which leads to internal hazards (Ghaly et al. 2018, 2022; Noli et al. 2021). Many physicochemical techniques have been developed for the decontamination of Cs-134 or retardation of its migration in the environment, such as precipitation/coprecipitation, reverse osmosis, ultrafiltration, adsorption, solvent extraction, and ion exchange either using synthetic or natural sorbents (Ahn et al. 2020; Lin et al. 2020; Noli et al. 2021; Şenol and Şimşek 2022). The sorption approach is widely used in the disposal of radionuclide-bearing wastewater due to its multiple superiorities of easy operation, low cost, high availability, and favorable removal performance (Veliscek-Carolan et al. 2013; Sankararamakrishnan et al. 2014; Karthik and Meenakshi 2015).

Scholars are particularly interested in the sorption of radionuclides onto natural clay minerals due to their abundant outputs, low cost, good thermal and mechanical stability, nontoxicity, eco-friendliness, and ion exchange capability (Zhang et al. 2019; Huang et al. 2020). Natural rocks and minerals can be used as adsorbent materials, such as bentonite, zeolite, and dolomite which are used by many researchers as adsorbents in the removal of Cs(I) ions from aqueous solutions due to their low cost (Belousov et al. 2019; Ibrahim et al. 2021; Şenol and Şimşek 2022). Bentonite is a natural aluminum phyllosilicate clay composed mainly of montmorillonite which belongs to the smectite group (Wan Ngah et al. 2011; Iwai and Hashimoto 2017; Wahab et al. 2019). The most characteristic property of the smectite group is their tendency to swell (i.e., expand their crystal structure) when reacted with aqueous and polar molecules by entering water or polar molecules in the interlayer structure. All the smectite group minerals are negatively charged on their external surface thus which describes their desirability to adsorb positively charged metal ions (Andrunik and Bajda 2019). The crystal structure of bentonite is composed of an octahedral alumina sheet (O) sandwiched between two contradicting tetrahedral (T) silicate sheets, 2:1 (T-O-T). The length of these sheets is several microns while the thickness is a few nanometers, and the gap in between is filled with exchangeable cations (Brigatti et al. 2013). Bentonite is widely used as a sorbent material for the decontamination of metal ions from wastewater because of its characteristic chemical and physical properties, (i.e., large specific surface area, high cation exchange capacity, and high desirability toward organic and inorganic ions) (Donat et al. 2005; Yang et al. 2014; Wahab et al. 2019; Şenol and Şimşek 2022).

There are many studies on the adsorption of Cs(I) ions in many inorganic sorbents such as Ti–Ca–Mg phosphates (Ivanets et al. 2020), sediment (Fuller et al. 2014), bentonites (Izosimova et al. 2022), and monetite (El-Din et al. 2018). But there were no studies carried out in the sorption of Cs(I) onto a novel MoV@bentonite composite that had not been prepared before. The use of modified bentonite was reported by (Olu-Owolabi and Unuabonah 2011; Dinh et al. 2022; Yang et al. 2022; Zhou et al. 2022). However, there are no reports on the adsorption capacity and the mechanism of MoV@bentonite as a sorbent material.

The novelty of this study is the decontamination of cesium-134 using MoV@bentonite which is a low-cost sorbent material with high reliability and performance. To overcome the negative feature of bentonite and to increase its interest in terms of adsorption, inorganic sorbent-bentonite composites are synthesized. The object of this study is to include the evaluation of a novel MoV@bentonite for cesium-134 decontamination from aqueous solutions. Different analytical tools were used to characterize the MoV@bentonite composite.

## Experiment

### Materials

Cesium chloride (CsCl, 99.99%), sodium vanadate (NaVO_3_, 98%), and sodium molybdate (Na_2_MoO_4_·2H_2_O, 99%) were supplied from Alpha Chemika, India; sodium tripolyphosphate (Na_5_P_3_O_10_, 95%) was supplied from Goway, China; nitric acid (HNO_3_, 65%) and hydrochloric acid (HCl, 35%) were supplied from Merck, Germany; and sodium hydroxide (NaOH, 99%) and ammonium hydroxide (NH_4_OH, 99%) were supplied from El-Nasr Co, Egypt. All chemicals and reagents used in this work were used without further purification. A stock solution of 1000 mg L^−1^ Cs(I) ions was prepared by dissolving the required amount CsCl in a definite volume of double distilled water.

### Radiotracer preparation

The second Egyptian training research reactor, ETRR-2 at Inshas, Egypt, was used to obtain ^134^Cs radioactive tracers. Specific grams of cesium chloride was wrapped in a thin thickness high-purity aluminum foil; then, it was held in a thick aluminum irradiation capsule after that the capsule was subjected to a pile of neutrons in the reactor. A definite quantity of the irradiated cesium chloride was dissolved in double distilled water. Equilibrium measurements were performed using the resulting isotopes as tracers. A single-channel analyzer supplied with a well-type NaI(Tl) detector was used to detect the γ-radiation radioactivity of the obtained isotopes.

### Preparation

**Fig. 1:**
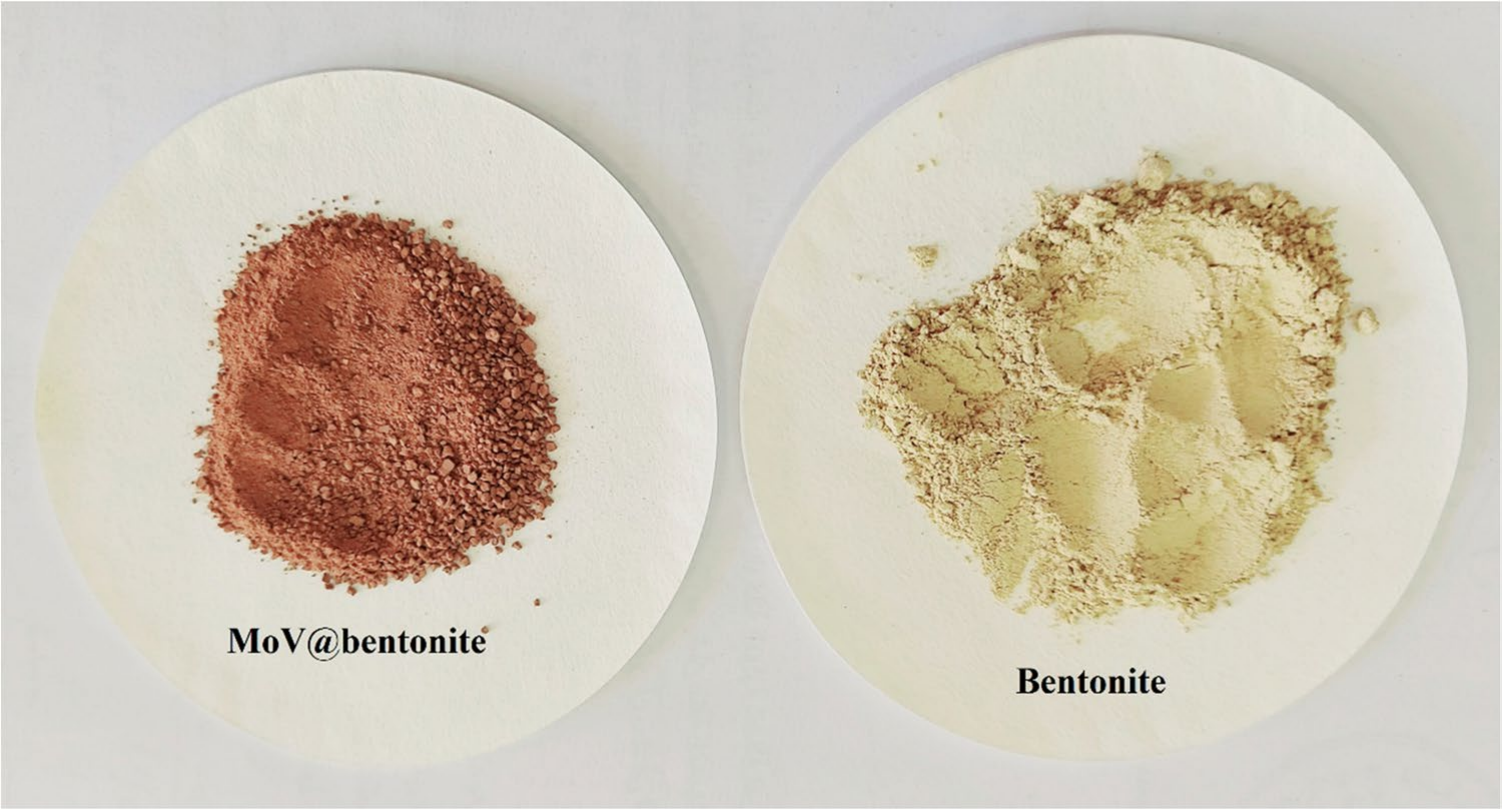
Picture of bentonite and MoV@bentonite composite

For the preparation of bentonite, molybdenum vanadate) MoV(, and MoV@bentonite composites, a simple and ambient precipitation procedure was used. The first stage is the preparation of bentonite solution by the addition of 2 g Na_5_P_3_O_10_ as a dispersing agent to 20 g bentonite ore with the addition of DDW to reach 200 mL with constant stirring for 120 min. The second stage is the preparation of 0.5 M Na_2_MoO_4_·2H_2_O and NaVO_3_ solutions by dissolving 12.1 g Na_2_MoO_4_·2H_2_O with 100 mL double distilled water and 6.097 g NaVO_3_ with 100 mL DDW. The third stage is where equimolar solutions (0.5 M) of Na_2_MoO_4_·2H_2_O and NaVO_3_ were added to bentonite solution with various volumetric ratios as illustrated in Table 1, after the component was added and stirred consistently for 2 h. After that, 10% ammonia solution was slowly added to mixtures with stirring to form a gel at a pH ranging from 7.5 to 8. Then, the reaction mixture was left undisturbed overnight, was washed several times with double distilled water, and was then dried at 60 °C. The resulting solid was converted into an H^+^ form by mixing it with 0.1 M HNO_3_ for 24 h, followed by filtering and multiple washed with double distilled water to remove the excess HNO_3_ and dried at 60 °C. Figure 1 demonstrates the prepared MoV@bentonite composite and ordinary bentonite. Transparent bentonite powder (yellow color) converted to brownish red color after the immobilization of MoV into the surface of the bentonite.

### Preliminary studies

The batch technique was used to study the sorption of Cs(I) ions onto bentonite or MoV or MoV@bentonite composites. Typically, 5 mL Cs(I) solution (50 mg·g^−1^) was added to 0.05 g of the synthesized composites and then shaken in a flask shaker for 24 h at 25 ± 1 °C. Hydrochloric acid and sodium hydroxide were used to adjust the pH of the solution. Finally, the radioactivity of the solution was measured and three replicates were prepared in each case. Equation (1) can be used to calculate the elimination efficiency (E%) (Hamed et al. 2019; Abass et al. 2021):

1 $$\mathrm{Elimination}\;\mathrm{efficiency}\;\left(\mathrm E\%\right)=\left(\frac{\mathrm{A}_{\mathrm i}-\mathrm{A}_{\mathrm f}}{\mathrm{A}_{\mathrm i}}\right)100$$

where *A*_*i*_ and *A*_*f*_ are the initial and final activity of ^134^Cs.

### Characterization

Fourier transform infrared (FT-IR) measurement was performed on a Nicolet is10 spectrometer (Meslo, USA) using KBr pellets for sample preparation. X-ray diffraction (XRD) was carried out by a Shimadzu XD-D1, X-ray diffractometer with Cu-Kα radiation tube source (*λ* = 0.15406 nm) and graphite monochromator operational at 30 kV and 30 mA. A scanning electron microscopy (SEM) and energy-dispersive X-ray (EDX) analysis model (ZEISS-EVO 15, UK) were utilized to examine the morphology and elemental analysis of the MoV@bentonite composite.

### Chemical stability study

The chemical stability of the MoV@bentonite composite was studied using different solvents (double distilled water, acetone, cyclohexanone, HNO_3_, HCl, and NaOH). Different solvents (20 mL) were added to MoV@bentonite composite (0.1 g) with constant agitation for about 3 days at room temperature. The amount of MoV@bentonite composite left in the solution was detected gravimetrically (Ibrahim et al. 2021; Kasem et al. 2021; Abass et al. 2022b, c).

### Batch studies

Different parameters such as pH (1–12), metal ion concentrations from 25 to 600 mg/L, sample weight (0.009–0.15 g), temperatures (298–338 K), and shaking time from 5 to 4320 min are examined carefully to obtain the optimum conditions for the sorption process. MoV@bentonite composite and Cs(I) ion solution were agitated batchwise till it reached equilibrium; the system was centrifuged at 6000 rpm to separate the liquid phase from the solid phase. In a shaker thermostat model (Kottermann D-1362, Germany), all equilibrium experiments were conducted by contacting 0.05 g of MoV@bentonite composite with 5 mL of Cs(I) ions of the initial concentration (*C*_*o*_) = 50 mg·g^−1^ with *V*/*m* = 0.1 L·g^−1^. The amount sorbed *q*_*e*_ (mg·g^−1^) was calculated from Eq. (2) (Metwally et al. 2019):

2 $${\mathrm q}_{\mathrm e\;}(\mathrm{mg}/\mathrm g)=({\mathrm C}_{\mathrm o}-{\mathrm C}_{\mathrm e})\frac{\mathrm V}{\mathrm m}$$

where *C*_*o*_ and *C*_*e*_ are the initial and equilibrium concentrations of Cs(I) ions in solution, *V* is the solution volume (L), and *m* is MoV@bentonite composite weight (g).

The distribution coefficients (*K*_d_) were computed from Eq. (3) (Şenol and Şimşek 2022):

3 $${\mathrm K}_{\mathrm d}\;(\mathrm{mL}/\mathrm g)=\frac{{\mathrm q}_{\mathrm e}}{{\mathrm C}_{\mathrm e}}$$

### Saturation capacity

The saturation capacity (*q*_*e*_) of bentonite and MoV@bentonite composites against Cs(I) ions was done by repeated equilibration of 50 mg·L^–1^ of Cs(I) ions at initial pH 12 with 0.05 g of adsorbent in a flask shaker at 25 ± 2 °C for 2 h. Each equilibration was continued for 5 h. The supernatant liquids were withdrawn, measured, and replaced by an equal volume of the original solution of respective ions. The equilibration was repeated until no further sorption of Cs(I) ions took place on synthesized sorbents. The saturated capacity was computed from Eq. (4).

4 $$\text{Saturation capacity=}\sum\limits_{\mathrm e=1}^{\mathrm n}{\mathrm q}_{\mathrm e}$$

where* n* is the sum number of times added new volumes.

### Desorption experiments

The desorption experiments were carried out to evaluate the efficiency of different eluents to release Cs(I) ions loaded on the synthesized composite. Desorption of Cs(I) ions from loaded MoV@bentonite was studied by using different eluents. The used eluents are CaCl_2_, HCl, and EDTA. A series of 50 mL bottles each containing 0.05 g of loaded MoV@bentonite by Cs(I) ions (50 mg·g^−1^) and 5 mL of (0.03, 0.05, and 0.1 M) from one of these eluents was shaken for 5 h. Finally, the suspensions were centrifuged and then analyzed in the supernatant (*C*_*s*_) and the solid phase (*C*_*d*_) (mg·g^−1^). The desorption percent was determined using Eq. (5) (Dakroury et al. 2021):

5 $$\%\;\mathrm{Desorption}=\left(\frac{{\mathrm C}_{\mathrm S}}{{\mathrm C}_{\mathrm d}}\right)100$$

## Results and Discussion

### Preliminary studies

The elimination efficiency of the studied radiotracer either onto bentonite or MoV or MoV@bentonite composites is presented in Table 1. Results reveal that the impregnation of MoV onto bentonite clay increases the elimination efficiency of MoV@bentonite (93.6%) than bentonite (82.6%) and MoV (81.5%); these results are due to increasing the number of active sites of MoV@bentonite leading to increasing in sorption behavior of ^134^Cs and MoV@bentonite which is the best adsorbent with the highest elimination efficiency of 93.6%.

**Table 1:** Conditions for the synthesis of bentonite, MoV, and MoV@bentonite composites and the percent removal of Cs(I) ions (50 mg/L and *V*/*m* = 100) at 30 ± 1 °C

Samples	Bentonite (10%)	NaVO_3_ (0.5 M)	Na_2_MoO_4_·2H_2_O (0.5 M)	The appearance of dried beads	E%
					^134^Cs
Bentonite	100	0	0	Yellowish gray	82.6
MoV	0	50	50	Yellowish gray	81.5
MoV@bentonite	100	50	50	Yellowish gray	93.6

### FT-IR analysis

**Fig. 2:**
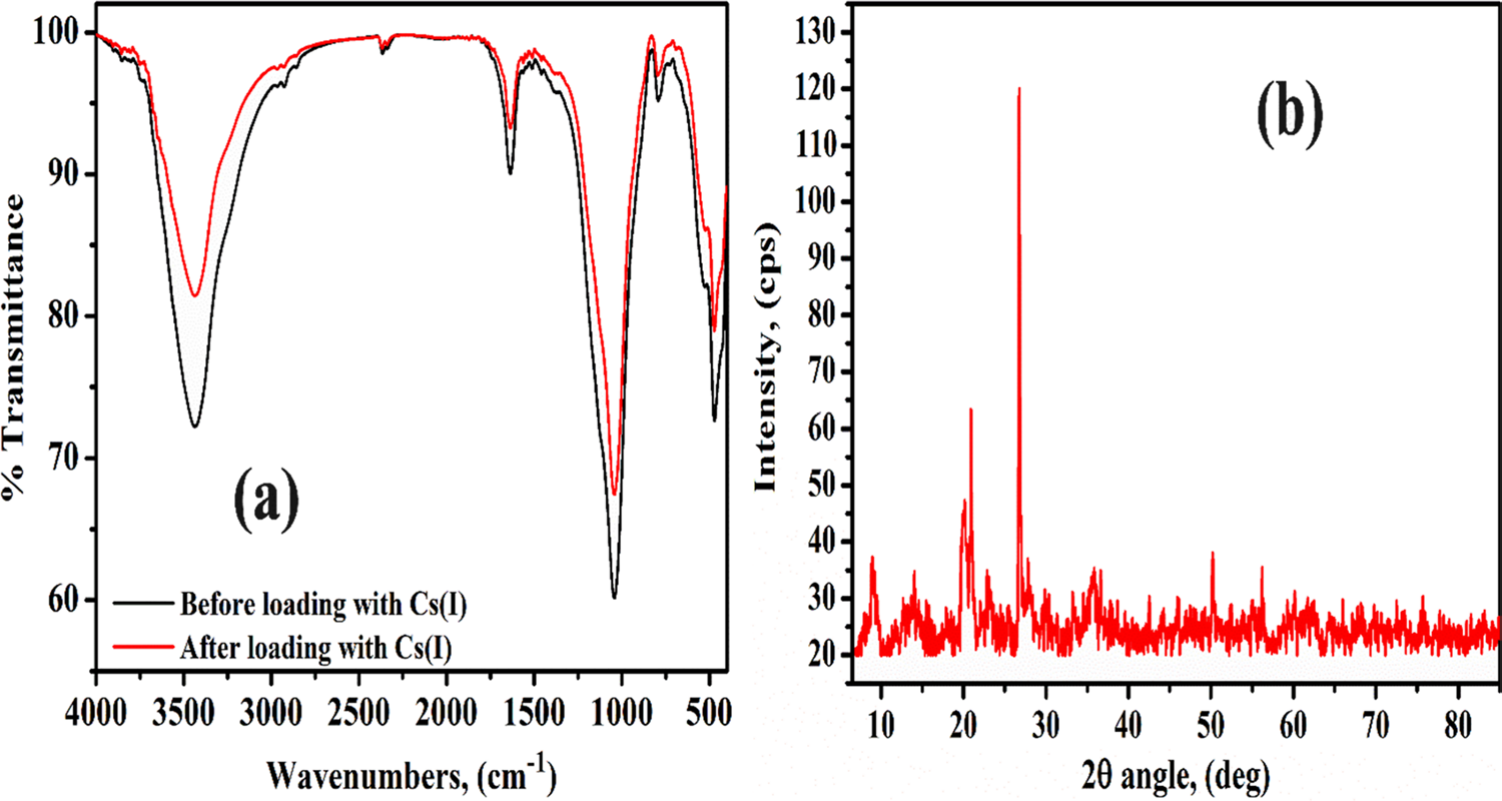
(**a**) FT-IR spectrum of MoV@bentonite composite before and after the sorption of Cs(I) ions and (**b**) XRD analysis of MoV@bentonite composite

FT-IR spectrum of MoV@bentonite before and after sorption of Cs(I) ions is shown in Fig. 2(a). The observed two bands located at 3437 and 1636 cm^−1^ can be explained by stretching and bending vibration of OH frequencies of intrastructure water molecules, respectively (Abass et al. 2022c) or attributed to V–OH groups (Rao et al. 2017; Xu et al. 2020). The band observed at 1511 cm^−1^ corresponds to M–O–H bending vibration mode (Abdel-Galil et al. 2020). Three bands observed at (1042, 792, and 526 cm^−1^) correspond to Si–O, Si–O-Al, and Si–O-Mg bending, respectively (Mekawy et al. 2022). The V–O bond vibrations in Mo–O–V matrix may be expressed by the FT-IR band at 471 cm^–1^ (Rao et al. 2017). FT-IR analysis shows that the spectrum of MoV@bentonite either before or after sorption of Cs(I) ions is approximately the same with a very slight shift except for the peak observed at 479 cm^−1^ which confirms the sorption of Cs(I) ions onto the surface of MoV@bentonite composite as seen later in EDX data. Moreover, the intensity of the band at 1042 cm^−1^ was increased which confirms the successful loading of Cs(I) ions onto the surface of the MoV@bentonite composite.

### XRD analysis

X-ray diffraction pattern of MoV@bentonite is exposed in Fig. 2(b). Several sharp peaks were located at 2Ɵ of (20.3°, 21.05°, 26.82°, 35.5°, and 50.1°) which confirms the crystalline nature of MoV@bentonite with a monoclinic system, where the detected peaks represent the Miller index indications (102, 201, 011, 400, and 412), respectively. These data are in agreement with the XRD of the BPN composite prepared by Mahrous et al. (Mahrous et al. 2022).

### SEM and EDX

**Fig. 3:**
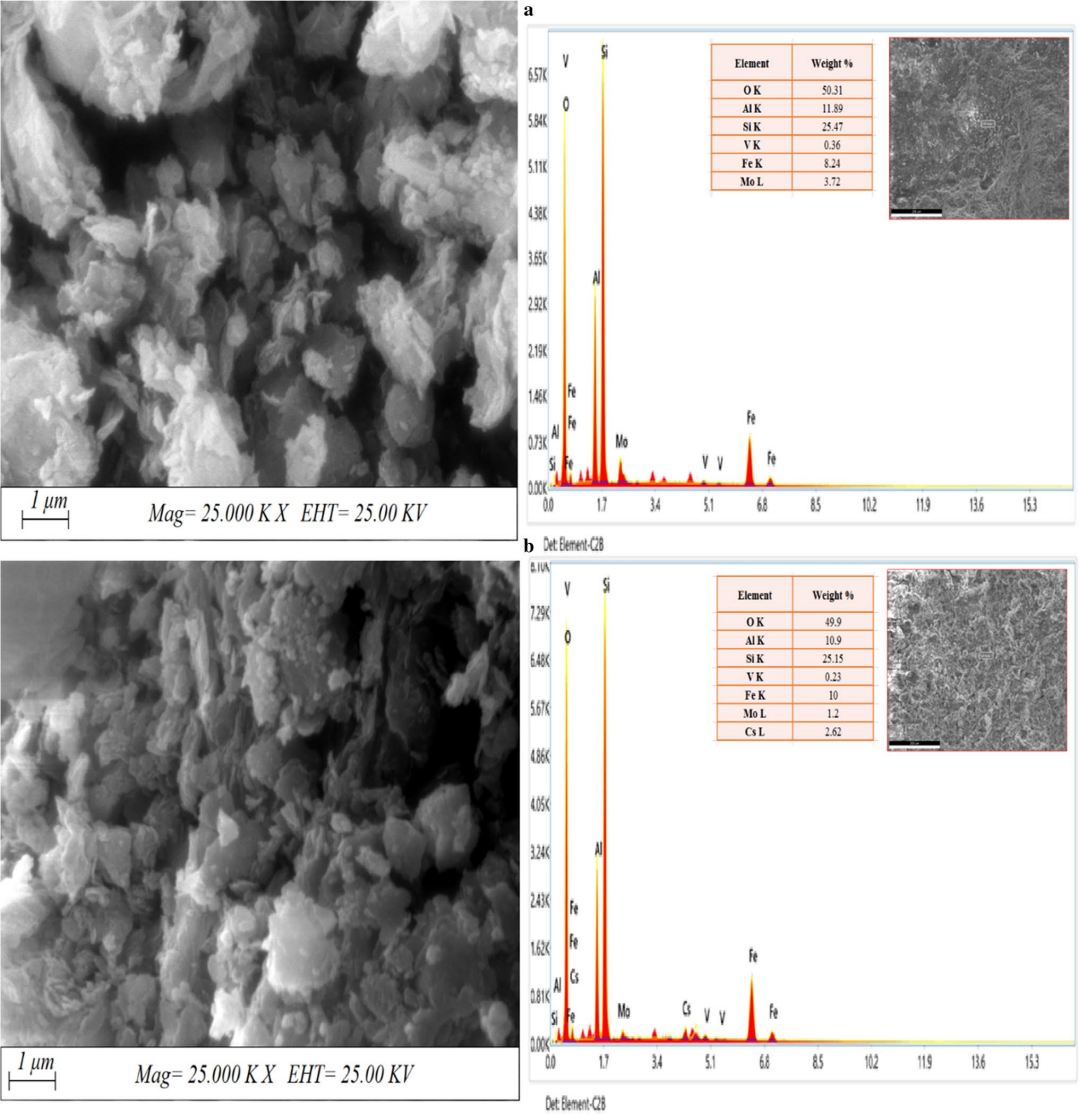
SEM-EDX of MoV@bentonite; (**a**) before and (**b**) after the sorption of Cs(I) ions

Figure 3 represents the morphology of the synthesized MoV@bentonite composite before and after the sorption of cesium ions. Figure 3(a) reveals the existence of a considerable number of small cracks and cavities which are filled with Cs(I) ions in Fig. 2(b). The elemental analysis of MoV@bentonite composite before and after sorption of Cs(I) ions is represented in Fig. 2. Results show that the percent of all the components is approximately the same except the percent of molybdenum which is reduced to be 1.2% instead of 3.72%; this reflects the exchange between Mo and Cs inside the structure of MoV@bentonite composite. This confirms that the modification of bentonite with molybdenum vanadate enhances the sorption properties of Cs(I) ions on it. This confirms that cesium ions can be sorbed either inside the cavities and cracks or exchanged with molybdenum.

### Chemical stability

The solubility test for MoV@bentonite toward different solvents (Table 2) indicated that the MoV@bentonite was very stable in different solvents which are mentioned in the experimental part. The data represented in Table 2 exhibit that MoV@bentonite composite has good chemical stability compared to other composites (Abdel-Galil et al. 2020, 2021; Ibrahim et al. 2021).

**Table 2:** Chemical stability for MoV@bentonite composite in different solvents

Solvents	Solubility (g/L)
DDW	Below detection
Acetone	Below detection
Cyclohexanone	0.065
1 mol/L HNO_3_	0.110
3 mol/L HNO_3_	0.176
1 mol/L HCl	0.146
3 mol/L HCl	0.219
0.5 mol/L NaOH	0.14
1 mol/L NaOH	0.185

### Optimization of batch studies

#### Influence of pH and point of zero charges

**Fig. 4:**
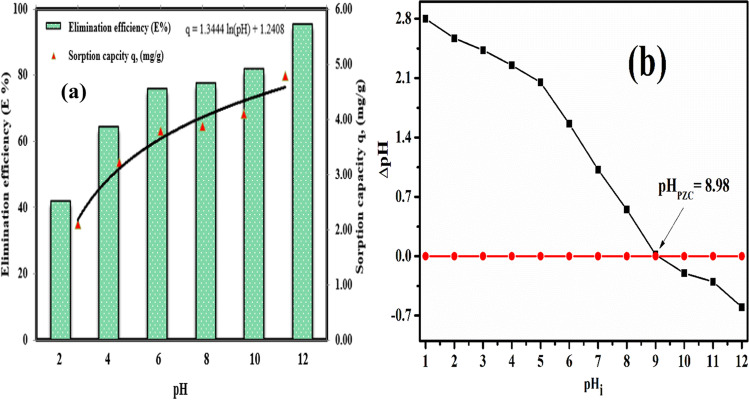
Sorption of Cs(I) ions onto MoV@bentonite; (**a**) effect of pH on the E% and sorption capacity and (**b**) PZC of MoV@bentonite

The adsorption process is highly affected by the solution pH, and the solution pH plays an important role in the adsorption process (Şenol and Şimşek 2022). Figure 4(a) shows the variation of the E% and equilibrium sorption capacity of Cs(I) ions onto MoV@bentonite composite, as a function of pH. The experiment was done at fixed temperature of 298 ± 1 K, *C*_*o*_ = 50 mg·g^−1^, *V*/*m* = 0.1 L·g^−1^, agitating time (300 min), and different pHs (1–12). It is evident that the values of E% and equilibrium sorption capacity of Cs(I) ions increase as the pH increases and it is seen that adsorption increases sharply as the pH increases from 2 to 6 and slightly increases from 8 to 12. It is seen that Cs(I) ion removal is lower at acidic pHs. This is because H^+^ ions and cationic Cs(I) ions compete to adsorb onto MoV@bentonite composite active centers. As the solution pH increases, the electrostatic repulsion forces between the Cs(I) ions and the MoV@bentonite composite surface decrease, increasing Cs(I) ion adsorption (Şenol and Şimşek 2022). At basic pHs, the surface of the MoV@bentonite composite is negatively charged and H-bonds and van der Waals interactions can be seen between the Cs(I) ions and the MoV@bentonite composite, so adsorption is seen to be high. The maximum E% was done at pH 12 with no change above this pH, and all experiments were done at this value.

The solution pH value at which the surface charge of the adsorbent is zero is defined as the point of zero charges (PZC). To determine the PZC value of the MoV@bentonite composite, the MoV@bentonite composite was kept in solutions containing 0.1 mol L^−1^ NaNO_3_ in the pH = 1–12 range for 24 h, and the equilibrium pH was measured. 0.1 M HCl or NaOH was used to adjust the pH. The PZC value was obtained from the linear relationship between the initial pH_i_ and ΔpH. The ΔpH value was obtained from the difference between the initial and final pH (ΔpH = pH_f_ − pH_i_). The surface charge of the MoV@bentonite composite was found to be 8.98 (Fig. 4(b)). The surface of the MoV@bentonite composite was positive (pH < pH_pzc_) below pH 8.98 and negative (pH > pH_pzc_) above pH 8.98. The condition pH > pH_pzc_ explains the increase.

#### Influence of shaking time

**Fig. 5:**
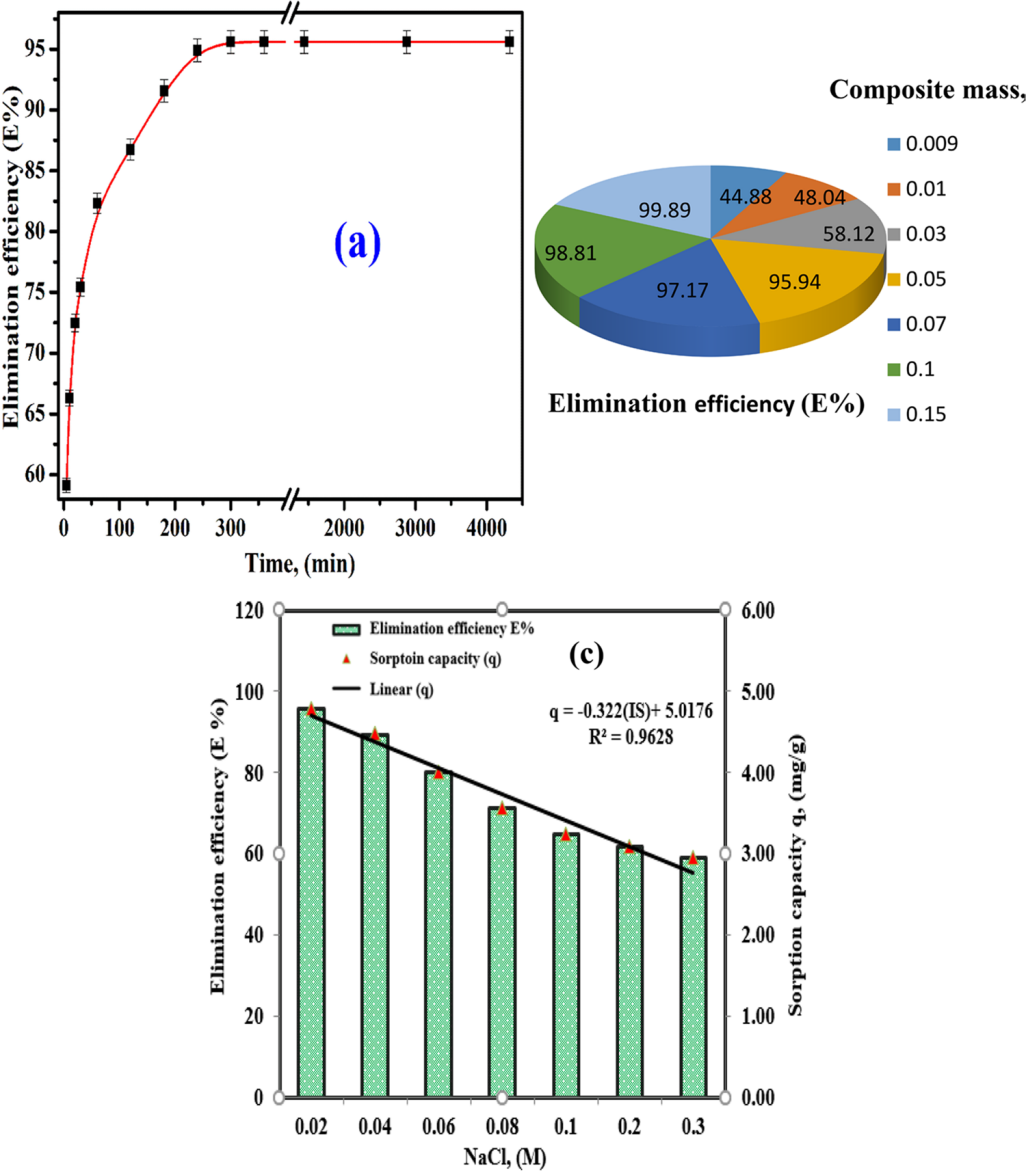
Sorption of Cs(I) ions onto MoV@bentonite at 298 ± 1 K; (**a**) effect of shaking time on E%, (**b**) effect of MoV@bentonite weight on E%, and (**c**) effect of ionic strength on the E% and sorption capacity

At fixed temperature (298 ± 1 K), initial concentration (*C*_*o*_) = 50 mg·g^−1^, *V*/*m* = 0.1 L.g^−1^, shaking time (5–4320 min), and pH = 12, the effect of shaking time on the elimination efficiency (E%) of Cs(I) ions onto MoV@bentonite composite is exposed in Fig. 5(a). It was found that the E% of Cs(I) ions onto MoV@bentonite composite very rapidly increases with time (5–180 min) and slowly increases (180–300 min), and there is no change of the E% after this time, so 300 min was used as equilibrium time.

#### Effect of MoV@bentonite dose

Fixed volume and different weights of MoV@bentonite composite were used to determine the minimal weight of MoV@bentonite composite to provide a reasonable E% of Cs(I) ions at a constant concentration. The weight of the synthesized composite was increased from 0.009 to 0.15 g, while the volume of the aqueous solution was fixed (5 mL). The effect of the MoV@bentonite weight on the E% of Cs(I) ions is shown in Fig. 5(b). The observations show that an increase in the E% occurs with the corresponding increases in the MoV@bentonite weight. The increases in the E% are due to the increase in its functional groups, and more active sites were available (Hamed et al. 2016b). As seen in Fig. 5(b), the E% of Cs(I) ions increased slowly from 44.8% to 58.1% when the MoV@bentonite weight was increased from 0.009 to approximately 0.03 g, whereas the sorption of Cs(I) ions increased quickly (58.1% to 95.93%) when the MoV@bentonite weight was changed from 0.03 to 0.05 g and then slightly (95.93% to 99.89%) when the MoV@bentonite weight increased from 0.05 g to 0.15 g.

#### Effect of ionic strength

Figure 5(c) shows the plots between the E% and sorption capacity of Cs(I) ions onto MoV@bentonite composite and the ionic strength of NaCl (0.02–0.3 M). The experiment was performed at *C*_*o*_ = 50 mg·g^−1^, *V*/*m* = 0.1 L·g^−1^, agitating time 300 min, and pH = 12. Figure 5(c) shows a great reduction in the E% of Cs(I) ions with increasing molar concentration of NaCl from 0.02 to 0.2 M. The E% of Cs(I) ions decreased from 82.69% to 51.15% with rising NaCl concentration up to 0.2 M. This reduction could be referred to as the presence of Na(I) retarded the movement of Cs(I) ions from bulk solution towards the surface of the MoV@bentonite composite. Ion exchange or outer-sphere surface complexation is primarily responsible for the strong ionic strength-dependent sorption (Sheha et al. 2020).

### Saturation capacity

The saturation capacity of Cs(I) ions on bentonite and MoV@bentonite composites was determined at 298 ± 1 K and pH 12 and recorded that 18.92 and 26.72 mg·g^−1^, respectively. The saturation capacity reveals that MoV@bentonite composite has a higher capacity value for Cs(I) ions than bentonite composite reflecting a good modification by adding MoV to bentonite composite as well as the MoV@bentonite composite has a higher capacity value for Cs(I) ions compared with inorganic composites (Yi et al. 2014; Jang et al. 2015; Hamed et al. 2016a; Abdel-Galil et al. 2019; Ke et al. 2020). Table 3 compares the capacity of MoV@bentonite composite with that of other composites for Cs(I) ions found in the literature. The prepared MoV@bentonite composite can be observed to have a higher capacity than many other sorbents. As a result, it can be seen that MoV@bentonite composite is a promising composite for removing Cs(I) ions from aqueous solutions.

**Table 3:** Comparison of capacities (mg/g) of Cs(I) ions on different composites under different conditions

Composites	^134^Cs	References
MoV@bentonite	26.72	Current work
Bentonite	18.92	Current work
SnSiMo	22.77	(Abdel-Galil et al. 2019)
Hydroxyl magnesium silicate	17.4	(Hamed et al. 2016a)
Copper ferrocyanide powder	15.2	(Ke et al. 2020)
Potassium titanium ferrocyanide	43.09	(Yi et al. 2014)
Prussian blue (PB)/reduced graphene oxide foam (RGOF)	18.67	(Jang et al. 2015)

### Kinetic study

Some kinetic equations such as pseudo-first-order (PFO), pseudo-second-order (PSO), and intra-particle diffusion model (Weber-Morris) (IPD) were used to check the experimental data and can be obtained from the next equations (Dao et al. 2022; Kumari et al. 2022; Şimşek et al. 2022):

6 $${\mathrm q}_{\mathrm t}={\mathrm q}_{\mathrm e}{(1-\mathrm e^{-{\mathrm K}_1\mathrm t})}^{\mathrm n}$$

7 $${\mathrm q}_{\mathrm t}=\frac{{\mathrm K}_2\mathrm q_{\mathrm e}^2\mathrm t}{1+{\mathrm K}_2{\mathrm q}_{\mathrm e}\mathrm t}$$

8 $${\mathrm q}_{\mathrm t}={\mathrm K}_\text{ipd}\;\mathrm t^{0.5}$$

**Fig. 6:**
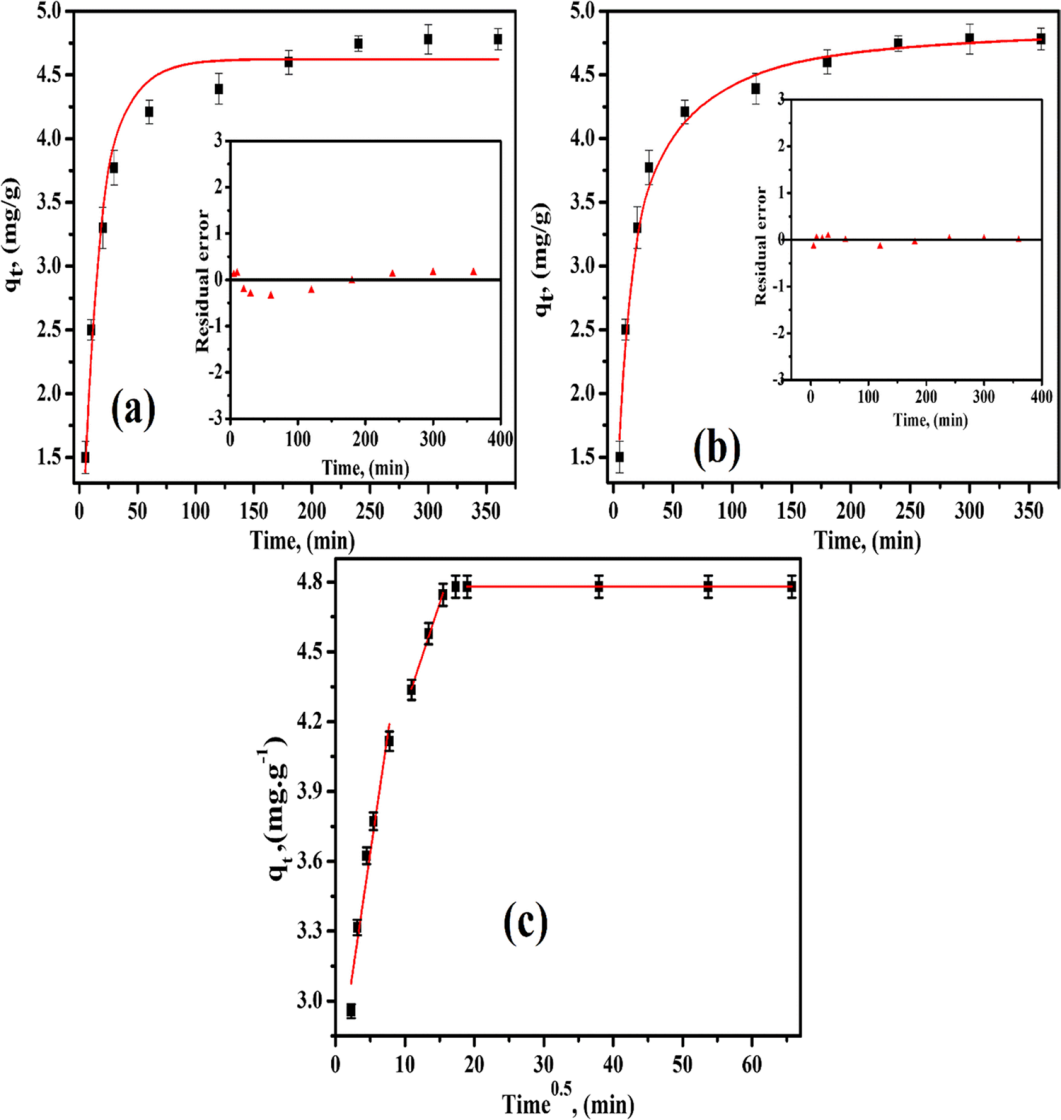
(**a**) PFO nonlinear fitting, (**b**) PSO nonlinear fitting, and (**c**) IPD of Cs(I) ions on MoV@bentonite at 298 ± 1 K [*C*_*o*_ = 50 mg/L, *V*/*m* = 0.1 L/g, pH = 12]

Here, *q*_*t*_ (mg·g^−1^) is the value of the amount sorbed per unit mass at any time *t*. *K*_1_ (min^−1^), *K*_2_ (g·mg^−1^·min^−1^), and *K*_IPD_ (mg/g·min^0.5^) are the rate constants of the PFO, PSO, and IPD, respectively. The plotted nonlinear forms of PFO and PSO are present in Fig. 6(a and b), respectively as well as the linear fitting form (IPD) as shown in Fig. 6(c). The *K*_1_, *K*_2_, and *K*_IPD_ constants as well as the correlation coefficient (*R*^2^) derived from these plots are presented in Table 4. In the first stage, rapid adsorption took place, involving the first 60 min. In the second phase (from 120 to 240 min), there was longer slower adsorption, possibly involving the interior of the adsorbent. The third stage from 300 to 4320 min includes the equilibrium saturation. The first stage was fast and quantitatively dominant, and the second stage was slower and quantitatively insignificant. During the initial stage of adsorption, there were many available active sites on the MoV@bentonite surface. After these sites were occupied, the equilibrium state was realized, and the second stage was started, involving the inner regions of the adsorbent. The rapid stage results from the abundance of active sites on the MoV@bentonite composite surface, while the gradual filling of these sites also makes the adsorption process less efficient during the slower stage. In the first stage of adsorption, there are many active sites on the MoV@bentonite composite surface. Cs(I) ions are adsorbed to these active sites. Over time, the number of active sites on the MoV@bentonite composite surface decreases, and the Cs(I) ions become saturated. In the next step, the Cs(I) ions diffuse slowly through the pore of the MoV@bentonite composite. Therefore, adsorption slows down. When the correlation coefficients (*R*^2^) of the PFO and PSO models were compared with each other, it was seen that the results fit the PSO kinetic model better. In addition, the closeness of the theoretically calculated q_t_ and experimental *q*_*e*_ values showed compatibility with the PSO model. These results showed that the adsorption process followed the PSO rate kinetics. Furthermore, the multilinear curves seen in the IPD plot indicated that the adsorption process involved two or more steps, as highlighted above. In this case, it was shown that it is not possible to explain the adsorption with a single kinetic model. Cs(I) ions first rapidly filled active sites on the surface of the MoV@bentonite composite and then diffused slowly and gradually through the pore of the MoV@bentonite composite. The adsorption process is accompanied by surface diffusion, film diffusion, diffusion on the pore surface, or more than one of these steps.

**Table 4:** Nonlinear fitting results of kinetic data using pseudo-first-order and pseudo-second-order as well as mass transport kinetic models for the sorption of Cs(I) ions onto MoV@bentonite composite

Pseudo-first-order		*q*_*e*_ (exp.) (mg·g^−1^)	Pseudo-second-order		
*q*_*e*_ (cal.) (mg·g^−1^)	4.62	4.78	*q*_*e*_ (cal.) (mg·g^−1^)		4.89
*K*_1_ (min^−1^)	0.07		*K*_2_ (g·mg^−1^·min^−1^)		0.26
*R*^2^	0.961		*R*^2^		0.963
Intraparticle diffusion					
Time range (min)	5–60	120–240		300–4320	
*K*_id_	0.202	0.090		–	
*R*^2^	0.943	0.994		–	

#### Effect of ion concentration

**Fig. 7:**
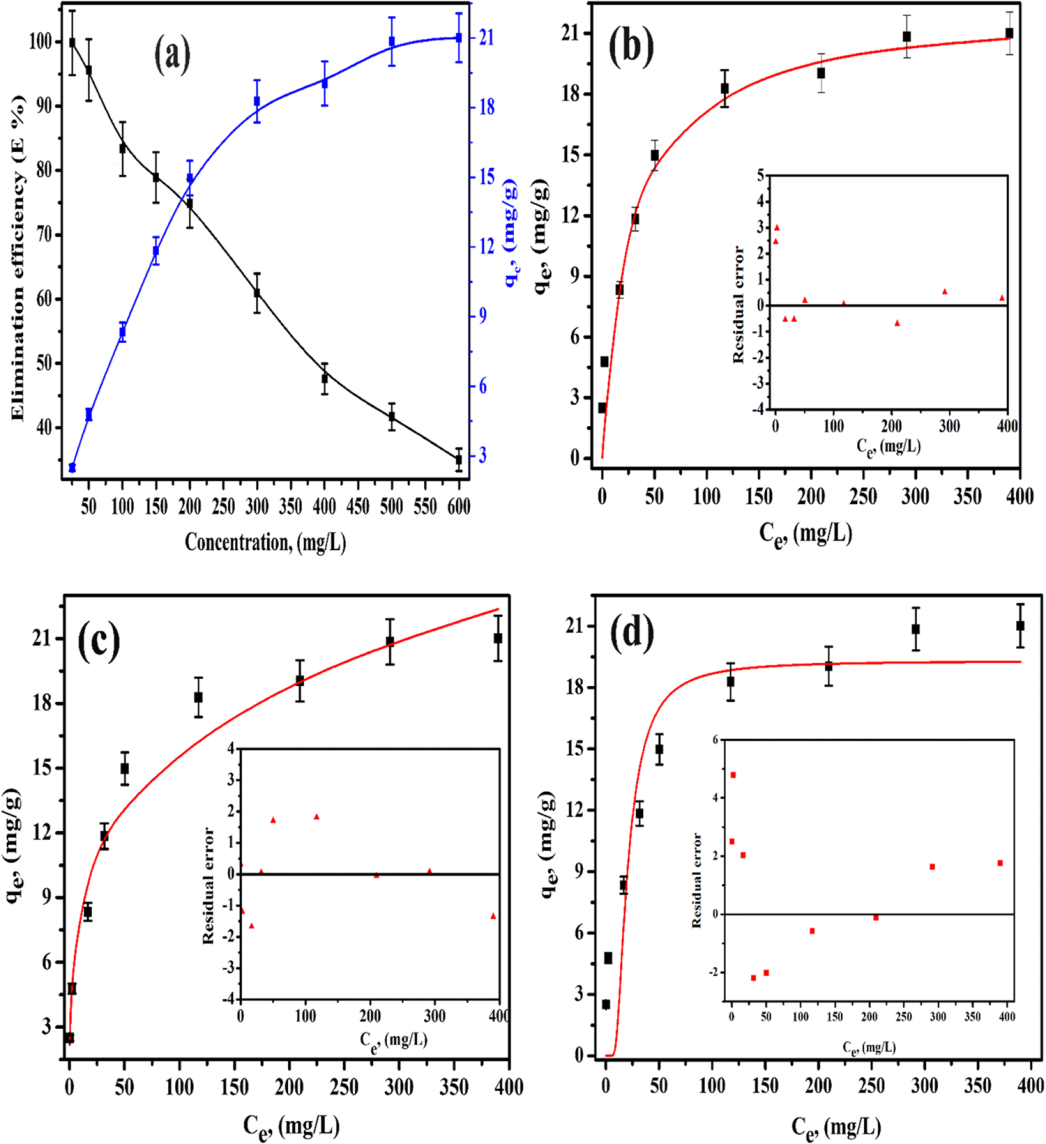
Sorption of Cs(I) ions onto MoV@bentonite at 298 ± 1 K; (**a**) effect of initial concentration on the E% and amount uptake, (**b**) nonlinear Langmuir plots, (**c**) nonlinear Freundlich plots, and (**d**) non-linear D-R plots

Figure 7(a) shows the plots between both E% and *q*_*e*_ (mg·g^−1^) of Cs(I) ions onto MoV@bentonite composite and the initial concentrations of Cs(I) ions. The experiment was performed at *C*_*o*_ = 25–600 mg/L, *V*/*m* = 0.1 L.g^−1^, agitating time of 300 min, and pH = 12. Figure 7(a) displays a great reduction in the E % of Cs(I) ions with increasing metal concentrations, and the maximum E % of Cs(I) ions was achieved at initial concentrations of 50 mg·g^−1^, whereas the amount uptake (mg·g^−1^) of Cs(I) ions increases with increasing initial concentration.

#### Sorption isotherm models

The concentration data obtained to determine the isotherms of the sorption of Cs(I) ions by MoV@bentonite composite were analyzed using Langmuir isotherm [Eq. (9)] (Chen et al. 2022), Freundlich isotherm [Eq. (10)] (Chang and Juang 2004), and Dubinin-Radushkevich (D-R) isotherm [Eqs. (11–13)] (Şenol et al. 2022) models. The Langmuir isotherm model assumes that the active sites where adsorption that took place is homogeneously distributed on the adsorbent surface (Langmuir 1918). The Freundlich isotherm model explains a hyperbolic adsorption behavior and gives information about the heterogeneity of the adsorbent surface (Freundlich 1907). The D-R isotherm model assumes that adsorption is related to surface porosity and pore volume. The D-R isotherm model examines adsorption from an energetic point of view and specifies the adsorption process physically or chemically. If the *E*_DR_ value is in the range of 8–16 kJ mol^−1^, the adsorption process is chemical; if the *E*_DR_ is < 8 kj mol^−1^, the adsorption process is physical. The *E*_DR_ value for this study was within the range of 8–16 kjmol^−1^ (Şenol et al. 2022).

9 $${\mathrm q}_{\mathrm e}=\frac{{\mathrm{bQ}}_{\max}{\mathrm C}_{\mathrm e}}{1+{\mathrm{bC}}_{\mathrm e}}$$

10 $${\mathrm q}_{\mathrm e}={\mathrm K}_{\mathrm F}\mathrm C_{\mathrm e}^\frac1{\mathrm n}$$

11 $${\mathrm q}_{\mathrm e}={\mathrm q}_{\max}\;\mathrm e^{-{\mathrm K}_{\mathrm{DR}^{\mathrm\varepsilon^2}}}$$

12 $$\mathrm\varepsilon=\mathrm{RT}\;\ln(1+\frac1{{\mathrm C}_{\mathrm e}})$$

13 $${\mathrm E}_\text{DR}=\frac1{\sqrt{2{\mathrm K}_\text{DR}}}$$

*Q*_max_ (mg·g^−1^) is the simulated monolayer capacity, and *b* (L/mg) is the Langmuir equilibrium constant related to the energy of sorption and describes the affinity of binding sites, *K*_*F*_ is Freundlich constant, $1/n$ is the adsorbent surface heterogeneity, *q*_max_ (mg·g^−1^) is a measure of adsorption capacity, *K*_DR_ and *ɛ* is based on sorption energy (Polanyi potential, kJ/mol), *R* is the ideal gas constant (8.314 Jmol^−1^ K^−1^), *E*_DR_ is the adsorption energy (kJ mol^− 1^), and *T* is the absolute temperature (K).

Adsorption isotherms were used to establish the interaction mechanism between adsorbent and Cs(I) ions at equilibrium. Comparing the *R*^2^ values derived from the Langmuir and Freundlich isotherm models (Fig. 7(b and c), Table 5), it can be seen that the adsorption process conformed to both Langmuir and Freundlich isotherm models. Sorption of Cs(I) ions onto MoV@bentonite composite provided a better fit with the Freundlich model (*R*^2^ = 0.96). *K*_*F*_, which is a measure of adsorption capacity, was 4.89, and 1/n surface heterogeneity was 0.26, from the Freundlich isotherm model. The 1/n surface heterogeneity showed that the conditions were favorable for the adsorption. The maximum adsorption capacity *Q*_max_ (mg·g^−1^) was 22.06 mg/g, and the *b* value was 0.04 L/mg. The high adsorption capacity of MoV@bentonite composite is promising for its use as an adsorbent for the removal of Cs(I) ions from aqueous solutions. The magnitude of the *E*_DR_ value in the D-R model characterizes the type of adsorption process (Fig. 7(d)). The adsorption energy, 14.21 kJ mol^−1^, derived by the D-R model, suggested that the nature of the adsorption is chemical.

**Table 5:** Isotherm parameters for sorption of Cs(I) ions onto MoV@bentonite composite

Langmuir			Freundlich			D-R		
*Q*_max_ (mg/g)	*b* (L/mg)	*R*^2^	1/*n*	*K*_*F*_ (mg/g)	*R*^2^	*q*_max_ (mg/g)	*E* (kJ/mol)	*R*^2^
22.06	0.04	0.952	0.26	4.89	0.96	21.23	14.21	0.87

### Thermodynamic studies

**Fig. 8:**
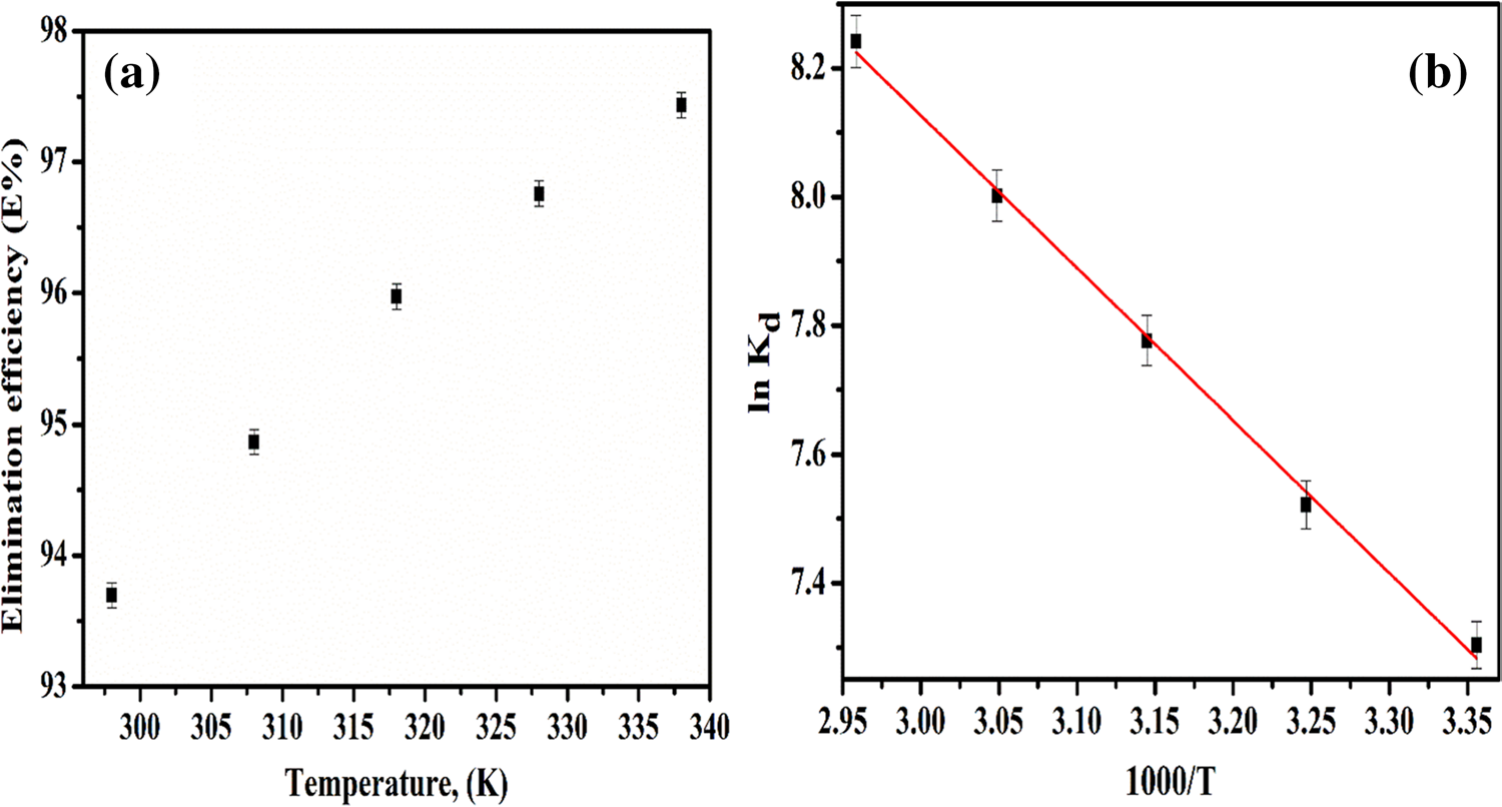
(**a**) Effect of reaction temperature on the E % of Cs(I) ions onto MoV@bentonite and (**b**) Van’t Hoff plot of the adsorption of ^134^Cs on MoV@bentonite composite

The influence of temperature on the E% of Cs(I) ions by MoV@bentonite composite is represented in Fig. 8(a). This figure illustrates how the endothermic nature of the sorption process is reflected by an increase in the E% of Cs(I) ions with increasing reaction temperature. The linear relationship between ln *K*_*d*_ of Cs(I) ions on MoV@bentonite composite and 1000/T through the Van't Hoff relation is shown in Fig. 7(b) (Abdel-Galil et al. 2016);

14 $$\ln\;{\mathrm K}_{\mathrm d}=\frac{\triangle\mathrm S^\circ}{\mathrm R}-\frac{\triangle\mathrm H^\circ}{\mathrm{RT}}$$

Here, $\Delta H^\circ , \Delta S^\circ , R, \mathrm{and}\;T$ are the changes of enthalpy (kJ/mol), change of entropy (J/mol·K), gas constant (J.K^−1^·mol^−1^), and absolute temperature (K). The *K*_*d*_ of Cs(I) ions improved with increasing reaction temperature from 298 to 338 °K. From both slopes and intercepts of linear relation shown in Fig. 7(b), $\Delta H^\circ$ and $\Delta S^\circ$ were computed and existing in Table 6. The positive values of both $\Delta H^\circ$ and $\Delta S^\circ$ reflect the endothermic nature and increased randomness of the solid solution interface through the adsorption of Cs(I) ions on MoV@bentonite composite, respectively (Abass et al. 2022c). The free energy change of adsorption ∆*G*° (kJ/mol) was obtained by the relation:

**Table 6:** Thermodynamic parameters for adsorption of Cs(I) ions onto MoV@bentonite composite

Temp. (K)	*∆H*° (kJ/mol)	*∆S*° (J/mol·K)	*∆G*° (kJ/mol)
298			− 18.05
308			− 19.31
318	19.70	126.7	− 20.58
328			− 21.85
338			
			− 23.11

15 $$\triangle\mathrm G^\circ=\triangle\mathrm H^\circ-\mathrm T\triangle\mathrm S^\circ$$

The negative values of ∆*G*° displayed in Table 6 reflect that the sorption process is spontaneous and indicates the better sorption of Cs(I) ions on MoV@bentonite composite compared with H^+^ ion (Abass et al. 2022c). By comparison of these data in the present work with other data from the literature, it is clear that ∆*H*° and ∆*S*° for Cs(I) sorbed onto MoV@bentonite composite have higher values than P(AN-AM)-NS and ZrSnP prepared by Abass et al. (Abass et al. 2022b, c) and lower than SnV prepared by Abass et al. (Abass et al. 2022a). And ∆*G*° for Cs(I) ions sorbed onto MoV@bentonite composite has lower values than P(AN-AM)-NS and ZrSnP prepared by Abass et al. (Abass et al. 2022b, c) and higher than SnV prepared by Abass et al. (Abass et al. 2022a). This comparison proves that the MoV@bentonite considers a promised composite for the sorption of Cs(I) ions.

### Adsorption mechanism

**Scheme 1:**
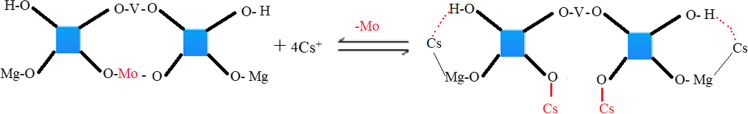
Sorption mechanism of Cs(I) ions onto MoV@bentonite composite

The mechanism of adsorption of Cs(I) ions onto the MoV@bentonite composite can be explained by the strong attraction forces of functional groups on the composite surface as shown in Scheme 1. The functional groups on the surface of the MoV@bentonite composite were mainly such as Mo–O, Mg-O, and hydroxyl groups (confirmed by the FT-IR spectrum). It could be very likely that electrostatic attraction plays an important role in the adsorption of Cs(I) ions. For a general mechanism, the following explanation was suggested: (i) migration of the Cs(I) ions onto the surface of the MoV@bentonite composite (bulk solution transport), (ii) diffusion of the Cs(I) ions across the boundary layer to the surface of the MoV@bentonite composite (film diffusion), and (iii) adsorption of the Cs(I) ions onto the surface of the MoV@bentonite composite. Mo–O, Mg-O, hydroxyl groups, and O bridges on the surface of MoV@bentonite composite form adsorption sites. The Mo–O and Mg-O may be exchanged with Cs(I) ions as seen in EDX results. The –OH groups of the MoV@bentonite composite could form H-bonds with the Cs(I) ions and function as adsorption sites. The final stage of the adsorption process could involve the penetration of some Cs(I) ions into the MoV@bentonite composite via intra-particle diffusion. In light of this information, it is thought that the adsorption process of Cs(I) ions in the MoV@bentonite composite are accompanied by surface diffusion, film diffusion, intraparticle diffusion, and diffusion on the pore surface.

### Desorption studies

**Fig. 9:**
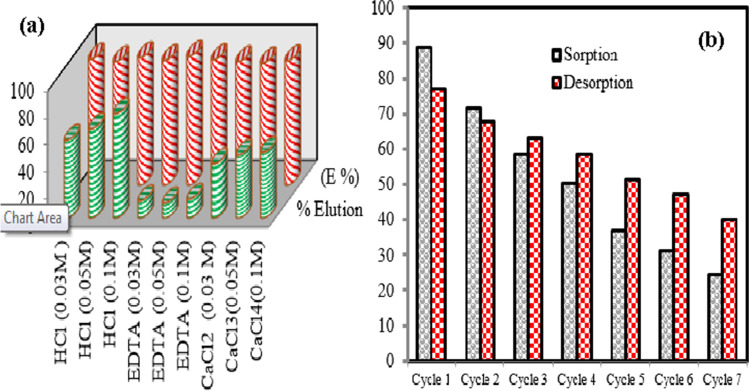
(**a**) Elimination efficiency (E %) and % elution of Cs(I) ions onto MoV@bentonite composite using different eluent agents and (**b**) recycling of MoV@bentonite composite for sorption of Cs(I) ions using 0.1 M HCl as an eluent

The desorption studies were carried out by eluting Cs(I) ions from loaded MoV@bentonite composite using different eluting agents such as CaCl_2_, HCl, and EDTA with different concentrations (0.03, 0.05, and 0.1 M), and the results are depicted in Fig. 9(a). Data illustrate that the recovery of studied radionuclide increase with increasing eluents concentration, and the best eluent is HCl (76.9%) at 0.1 M. The sequence order for the % desorption process is HCl ˃ CaCl_2_ ˃ EDTA.

### Recycling study

The repeated use of MoV@bentonite composite is an essential feature where the subsequent regeneration of the sorbent for another cycle of the application is desirable. The recycling efficiency of MoV@bentonite composite and its potential use as a solid phase was done by successive adsorption and desorption cycles of Cs(I) ions at the optimal condition. MoV@bentonite composite loaded with Cs(I) ions was recycled using 0.1 M HCl as an eluent. This eluent is used because it attained the highest desorption percentage of Cs(I) ions from the loaded-on MoV@bentonite composite in the desorption experiment compared with the other used eluents. The synthesized MoV@bentonite composite was used in repeated sorption–desorption cycles. Figure 9(b) illustrates the relationship between the E% and the cycle number. The result illustrated that the synthesized MoV@bentonite composite could be reused for sorption–desorption cycles up to 7 cycles with a slight decrease in the E% from cycle one to cycle two also, the E% slightly decreased from cycle two to cycle three, and so on. The excellent regeneration argues the applicability of MoV@bentonite to be used repeatedly as an effective material for the sorption of Cs(I) ions from aqueous media with very good efficiencies.

## Conclusion

In this article, the MoV@bentonite composite was successfully synthesized through the precipitation method and tested for its performance in the sorption of Cs(I) ions from aqueous solutions. The sorption experiments of Cs(I) ions confirm that the prepared composite has an equilibrium time of (300 min), applicable to the Langmuir and Freundlich isotherm models, and more fitted to the pseudo-second-order kinetic model. Experiments show that the sorption of Cs(I) ions onto MoV@bentonite composite is dependent on pH values and the optimum pH value is pH = 12. Saturation capacity experiments illustrate that the maximum Cs(I) ions sorbed onto MoV@bentonite composite is 26.72 mg·g^−1^. Thermodynamic parameters were endothermic and spontaneous. 0.1 M HCl shows optimal desorption of Cs(I) ions from an aqueous solution. All the previous data show that MoV@bentonite composite has several advantages such as low cost, high reliability, and high performance for decontamination of cesium-134 while the chief disadvantage is that the equilibrium was reached within 300 min. Finally, the high sorption efficiency of the MoV@bentonite composite suggests that it can be used as an effective and promising sorbent for Cs(I) ions from aqueous solutions in the future.

## Data Availability

Yes.
